# Unveiling the potential pleiotropic effects of metformin in treating COVID-19: a comprehensive review

**DOI:** 10.3389/fmolb.2023.1260633

**Published:** 2023-10-10

**Authors:** Pavlo Petakh, Iryna Kamyshna, Aleksandr Kamyshnyi

**Affiliations:** ^1^ Department of Biochemistry and Pharmacology, Uzhhorod National University, Uzhhorod, Ukraine; ^2^ Department of Microbiology, Virology, and Immunology, I. Horbachevsky Ternopil National Medical University, Ternopil, Ukraine; ^3^ Department of Medical Rehabilitation, I. Horbachevsky Ternopil National Medical University, Ternopil, Ukraine

**Keywords:** gut microbiota, coronavirus disease 2019, dysbiosis, metformin, diabetes

## Abstract

This review article explores the potential of metformin, a medication commonly used for type 2 diabetes, as an antiviral and anti-inflammatory agent in the context of coronavirus disease 2019 (COVID-19). Metformin has demonstrated inhibitory effects on the growth of SARS-CoV-2 in cell culture models and has shown promising results in reducing viral load and achieving undetectable viral levels in clinical trials. Additionally, metformin exhibits anti-inflammatory properties by reducing the production of pro-inflammatory cytokines and modulating immune cell function, which may help prevent cytokine storms associated with severe COVID-19. The drug’s ability to regulate the balance between pro-inflammatory Th17 cells and anti-inflammatory Treg cells suggests its potential in mitigating inflammation and restoring T cell functionality. Furthermore, metformin’s modulation of the gut microbiota, particularly changes in bacterial taxa and the production of short-chain fatty acids, may contribute to its therapeutic effects. The interplay between metformin, bile acids, the gut microbiome, glucagon-like peptide-1 secretion, and glycemic control has implications for the management of diabetes and potential interventions in COVID-19. By refreshing the current evidence, this review highlights the potential of metformin as a therapeutic option in the management of COVID-19, while also exploring its effects on the gut microbiome and immunometabolism.

## Introduction

Coronavirus disease 2019 (COVID-19), a global pandemic caused by the novel coronavirus Severe Acute Respiratory Syndrome Coronavirus 2 (SARS-CoV-2), has impacted millions of individuals across the globe (Atzrodt et al., 2020). People with pre-existing conditions like type 2 diabetes (T2D) are at a higher risk of experiencing severe outcomes from COVID-19 (Al-Kuraishy et al., 2021; Kamyshnyi et al., 2021). T2D is a chronic metabolic disorder characterized by insulin resistance and elevated blood glucose levels, which can lead to various complications and impaired immune function (Hameed et al., 2015).

Metformin, a commonly prescribed medication for T2D, has garnered attention for its potential benefits beyond glycemic control (Kamyshnyi et al., 2021). Recent research indicates that metformin may possess antiviral and anti-inflammatory properties, making it a promising candidate for combating SARS-CoV-2 infection and reducing the adverse effects of COVID-19 (Petakh et al., 2022a; Petakh et al., 2022b; Petakh et al., 2022c; Petakh et al., 2023a; Petakh et al., 2023b; Petakh et al., 2023c; Petakh et al., 2023d; Erickson et al., 2023).

This review article aims to present an overview of the current evidence related to metformin’s potential anti-SARS-CoV-2 effects, its anti-inflammatory properties, and its role in modulating the Th17/Treg balance. Additionally, it delves into the interactions between metformin, the gut microbiota, bile acids, and glycemic control concerning COVID-19 and T2D.

## Anti-SARS-CoV-2 effect of metformin

Metformin, an established medication for T2D, has recently gained attention for its potential antiviral properties against SARS-CoV-2, as well as other RNA viruses such as Zika, dengue, hepatitis B, hepatitis C, influenza, and human immunodeficiency viruses (HIV) (Farfan-Morales et al., 2021; Justice et al., 2021; Wiernsperger et al., 2022). Early studies in the 1940s even demonstrated the beneficial effects of metformin in treating patients with influenza (Ibrahim et al., 2021). In the context of SARS-CoV-2, metformin may exert its antiviral effects by preventing viral entry into cells through conformational changes in the angiotensin I converting enzyme 2 (ACE2) receptor, which is thought to be facilitated by AMP-activated protein kinase (AMPK)-mediated phosphorylation at S680 of the ACE2 protein (Sharma et al., 2020).

Additionally, metformin’s impact on intracellular pH regulation within endosomes is noteworthy. Key components involved in pH regulation within endosomes, such as Vacuolar ATPase (V-ATPase) and endosomal Na^+^/H^+^ exchangers (eNHE), play critical roles in this process. Research has indicated that metformin can directly impact eNHE and/or V-ATPase, leading to elevated pH levels within endosomes. This elevation in pH levels may suppress viral infection (Esam, 2020; Eaton et al., 2021). Moreover, metformin might also have the potential to prevent the development of pulmonary fibrosis, a complication associated with COVID-19 (Esam, 2020).

**FIGURE 1:**
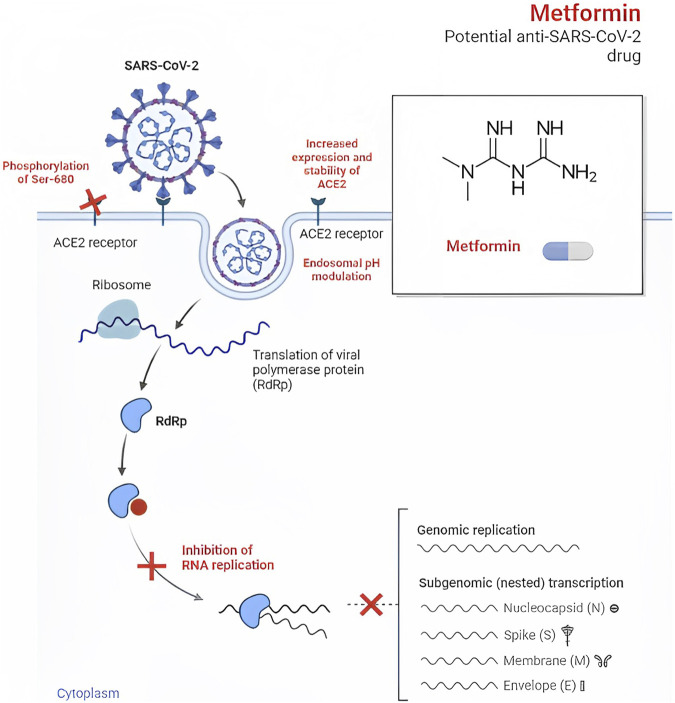
Effects against SARS-CoV-2.

In a study conducted by Haripriya Parthasarathy et al., metformin’s significant inhibitory effect on the growth of SARS-CoV-2 was demonstrated in cell culture models (Parthasarathy et al., 2022). As the viral infection progressed, AMPK phosphorylation showed a steady increase, indicating its crucial role during the infection process. When AMPK was activated in Calu3 and Caco2 cell lines using metformin, there was a remarkable suppression of SARS-CoV-2 infectious titers, with up to 99% reduction observed in infected cells. Dose-variation studies revealed half maximal inhibitory concentration (IC_50_) values of 0.4 mM and 1.43 mM in Calu3 and Caco2 cells, respectively. The involvement of AMPK in metformin’s antiviral effect was further confirmed by using other pharmacological compounds such as 5-Aminoimidazole-4-carboxamide ribonucleoside (AICAR) and Compound C (Table 1; Figure 1).

**TABLE 1:** Examination of the impact of metformin on SARS-CoV-2 infection models.

Author, year	Study design	Substrate	Key findings
Bramante et al. (2023)	*In vivo* (randomized trial)	COVID-19 patients	Metformin demonstrated a possible benefit in preventing more severe outcomes such as emergency department visits, hospitalization, or death
Carolyn et al. (2023)	*In vivo* (randomized trial)	COVID-19 patients	Metformin showed a 42% reduction in ER visits/hospitalizations/death through 14 days and a 58% reduction in hospitalizations/death through 28 days. Furthermore, metformin demonstrated a 42% reduction in Long COVID through 10 months. Viral load analysis revealed a 3.6-fold reduction with metformin compared to placebo
Sun et al. (2022b)	*In vitro* experimental study	Human airway epithelial cell lines (BEAS2B, A549, and 16HBE)	Metformin exhibited multifaceted effects, including restoration of autophagy, suppression of pyroptosis, and attenuation of inflammatory response
Parthasarathy et al. (2022)	*In vitro* experimental study	Calu3 (respiratory epithelial cell line) and Caco2 (gut epithelial cell line)	Metformin pretreatment effectively suppressed viral replication and protein expression in both respiratory and gut epithelial cell lines
Ventura-López et al. (2022)	*In vitro* experimental study	H1299 and Vero E6 cell lines	Metformin glycinate demonstrated significant reduction in viral load and enhanced cell viability against different SARS-CoV-2 variants
Mercado-Gómez et al. (2022)	*In vitro* experimental study	Human primary hepatocytes, human upcyte second-generation hepatocytes, humanized ACE2 (hACE2) mice, and wild-type mice	Metformin exhibited hepatoprotective effects by suppressing ACE2 expression, reducing viral infection rates, and modulating inflammatory markers in hepatocytes
Cory et al. (2021)	*In vitro* experimental study	Purified classical monocytes from healthy human subjects	Metformin pretreatment resulted in the suppression of glycolytic response and downregulation of pro-inflammatory cytokines upon viral exposure
Chen et al. (2021)	*In vitro* and *in vivo* assays	*In vivo*: Midbrain dopaminergic neurons derived from H9 human embryonic stem cells injected into mice. *In vitro*: Midbrain dopaminergic neuron cell line derived from human pluripotent stem cells	Metformin exhibited antiviral effects by reducing viral RNA levels and preventing cellular senescence in midbrain dopaminergic neurons
Schaller et al. (2021)	*Ex vivo* and *in vitro* assays	Cryopreserved bank of human lung tissue and Vero E6 cell line	Metformin exhibited variable efficacy, with significant reduction in SARS-CoV-2 titers observed in lung tissues but not in Vero E6 cell line
Xian et al. (2021)	*In vitro* and *in vivo* assays	Bone marrow–derived macrophages from nondiabetic mice	Metformin displayed immunomodulatory effects by inhibiting NLRP3 inflammasome activation, cytokine production, and mitochondrial dysfunction
Gordon et al. (2020)	*In vitro* experimental study	Vero E6 cell line	Metformin demonstrated potent antiviral activity by inhibiting viral replication and promoting cellular viability

Additionally, promising results were observed in the viral load analysis from a clinical trial (Carolyn et al., 2023). The mean change in viral load from baseline to follow-up was notably lower with metformin compared to the placebo group (−0.64 log_10_ copies/mL), indicating a 4.4-fold greater decrease in viral load. On day 5, the rate of undetectable SARS-CoV-2 viral load was 49.9% in the metformin group and 54.6% in the placebo group, with a modest odds ratio of 1.235. Similarly, on day 10, the undetectable rate was 14.3% in the metformin group and 22.6% in the placebo group, with an odds ratio of 1.663, demonstrating a statistically significant difference between the groups.

Ventura-López et al. conducted a trial where metformin treatment demonstrated substantial advantages compared to the placebo group. The metformin-treated participants experienced a significant decrease in the need for supplemental oxygen, a more pronounced reduction in the percentage of viral load, and a quicker attainment of an undetectable viral load. However, there were no significant differences in the duration of hospitalization between the metformin and placebo groups (Ventura-López et al., 2022).

In the TOGETHER Trial, the use of metformin did not yield a significant decrease in hospitalizations due to COVID-19. Hospitalization was defined as either remaining in a COVID-19 emergency setting for more than 6 h or being transferred to a tertiary care hospital within 28 days after randomization. The statistical analysis showed that there was no significant difference between the metformin and placebo group, with a relative risk of 1.14 and a 95% credible interval of 0.73–1.81. Additionally, metformin did not significantly affect viral clearance at day 7, time to hospitalization, or clinical improvement 28 days after randomization. However, when considering the per-protocol sample, which accounted for 83% of the participants, there was a reduced likelihood of emergency department visits and hospitalizations of COVID-19 patients, resulting in an absolute risk reduction of 1.4% and 3.1%, respectively (Reis et al., 2022).

## Interferon responses to SARS-CoV-2 and SAMHD1-cGAS-STING-metformin interactions

Sterile alpha motif and histidine-aspartate domain-containing protein 1 (SAMHD1) negatively regulates the interferon −1 signaling pathway: the elevated innate immune response and IFN activation upon genetic loss of SAMHD1 effectively suppress SARS-CoV-2 replication (Oo et al., 2022).

The cGAS-STING pathway is central to detecting viral DNA and initiating immune responses. Activation of cGAS-STING triggers IRF3-mediated type I IFN production and autophagy-mediated antiviral activity. cGAS produces cyclic GMP-AMP (cGAMP), activating STING (Su et al., 2023). This pathway responds to viral and bacterial DNA, as well as self-DNA from senescent or dying cells.

SARS-CoV-2 infection induces micronuclei formation, activating the cGAS-STING pathway, which can damage cells. Viral proteins like ORF3a and ORF10 can interfere with this pathway to evade immune responses (Han et al., 2022).

Metformin, a versatile drug, has significant effects on cGAS-STING signaling. For example, in gastric cancer, metformin promotes cGAS/STING activation by inhibiting AKT phosphorylation (Shen et al., 2023). It also activates type I IFN signaling against hepatitis C virus through AMPK (Tsai et al., 2017).

However, it is important to note that metformin can have inhibitory effects on the type I IFN response in specific immune cells, like human CD4^+^ T cells (Titov et al., 2019). This complex interplay between metformin, the cGAS-STING pathway, and type I IFN signaling underscores the intricate mechanisms involved in the immune response against viral infections, including SARS-CoV-2.

## Anti-inflammatory effect of metformin: a protective mechanism against cytokine storm

Metformin exerts its effects through various mechanisms, including the activation of liver kinase B1, which leads to the activation of AMPK, as well as the activation of NAD-dependent deacetylase sirtuin-1 (SIRT1) and peroxisome proliferator–activated receptor γ coactivator-1 α (PGC-1α) (Kulkarni et al., 2020; Triggle et al., 2022). Furthermore, metformin inhibits mitochondrial complex 1, nuclear factor κ-light-chain-enhancer of activated B cells (NF-κB), and mammalian target of rapamycin complex 1 (mTORC1) (Kulkarni et al., 2020).

Metformin possesses anti-inflammatory properties, beneficial for individuals both with and without diabetes (Cameron et al., 2016). In the context of COVID-19, its anti-inflammatory actions involve reducing the levels of various pro-inflammatory factors, including tumor necrosis factor-α (TNF-α), interleukin-1β (IL-1β), interleukin-6 (IL-6), C-X-C motif chemokine ligand 5 (CXCL5), C-X-C motif chemokine ligand 10 (CXCL10), and monocyte chemoattractant protein-1 (MCP-1) (Justice et al., 2021). Moreover, metformin inhibits the signal transducer and activator of transcription 3 (STAT3) and has been shown to decrease the formation of neutrophil extracellular traps (Bailey and Gwilt, 2022). Another potential effect of metformin is preventing the activation of the NLR family pyrin domain-containing 3 (NLRP3) inflammasome (Wiernsperger et al., 2022).

Observational data suggest that prior usage of metformin before COVID-19 infection is associated with lower levels of peak C-reactive protein (CRP), as well as lower rates of admission and peak ferritin in a subgroup analysis of patients in the intensive care unit (Ma et al., 2022).

## Metformin modulation of Th17/Treg balance: anti-inflammatory effects. Targeting immunometabolism to prevent cytokine storm in COVID-19

**FIGURE 2:**
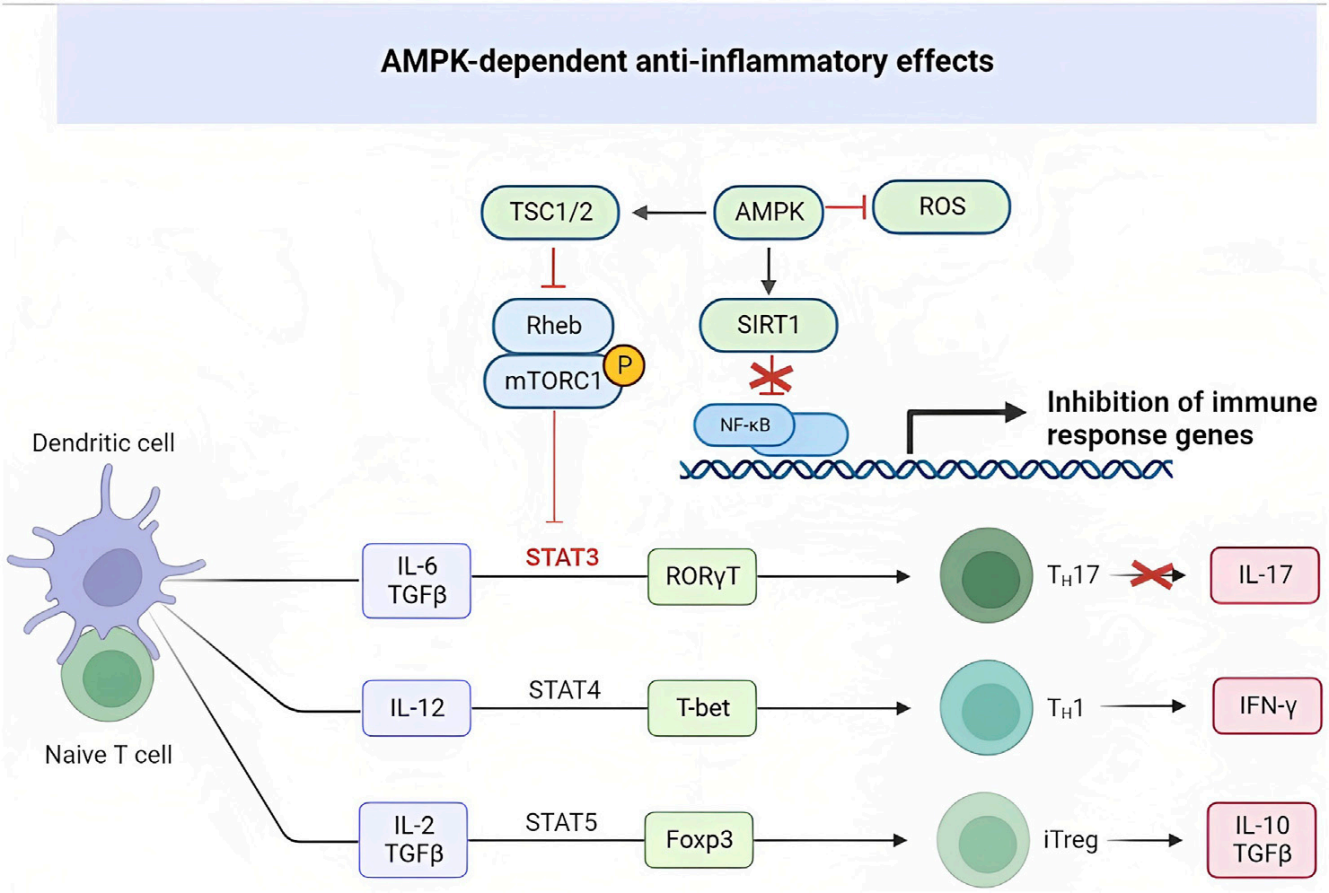
AMPK-dependent anti-inflammatory effects.

Metformin has been shown to affect Th17 cell differentiation through the AMPK/mTOR/STAT3 pathway (Tan et al., 2019). In diseases like COVID-19 and systemic lupus erythematosus (SLE), there is an increase in Th17 cells (Duan et al., 2019). Metformin’s influence on CD4^+^ T cell glucose metabolism, achieved by inhibiting mitochondrial complex I and oxidative phosphorylation, helps normalize cellular processes crucial for CD4^+^ T cell activation, proliferation, and differentiation. By targeting overactive effector T cells, including Th1 and Th17 cells, as well as proinflammatory cytokines such as interferon interferon-gamma (IFN-γ) and IL-17, metformin shows potential in reducing inflammation in Systemic Lupus Erythematosus (SLE) (Tan et al., 2019). In viral infections such as COVID-19, CD8^+^ T cells play a vital role in eliminating the virus by releasing cytotoxic molecules like perforin, granzyme, and IFN-γ (Omarjee et al., 2020). The use of metformin at the doses prescribed for diabetes treatment could potentially help restore T cell functionality and alleviate the cytokine storm commonly observed in COVID-19 (Salvatore et al., 2020; Scheen, 2020; Sharma et al., 2020) (Figure 2).

AMPK and mTOR serve as metabolic detectors that influence the balance between pro-inflammatory and anti-inflammatory cells. Th17 cells rely on glycolysis for their energy needs, while regulatory T cells (Tregs) rely on fatty acid oxidation. AMPK’s ability to regulate metabolism affects the differentiation of these cell types. On the other hand, mTOR activation, leading to the induction of HIF1α, promotes glucose import and glycolysis at the transcriptional and translational level. Lack of HIF1α induction significantly reduces Th17 cells. In a rat model of collagen-induced arthritis (CIA), metformin was found to activate AMPK, inhibit mTOR, and regulate the Th17/Treg ratio. Metformin also reduced the levels of proinflammatory cytokines TNFα, IL-1β, IL-6, and IL-17 in the serum of rats with CIA, while decreasing the number of splenic CD4+/RORγt+/IL-17 + T cells (Th17s) in a dose-dependent manner. Furthermore, metformin positively correlated with the increase of regulatory T cells (CD4+/CD25+/FOXP3+) in the study (Yang et al., 2017). Likewise, metformin played a role in alleviating autoimmune insulitis in a model of Type 1 diabetes (T1D) using NOD mice. Female NOD mice were administered metformin or a control substance starting at 4 weeks of age. By the time the mice reached 12 weeks of age, they showed signs of insulitis. However, the mice treated with metformin displayed a higher number of functional β cells compared to those on the control treatment. Additionally, metformin significantly reduced the number of pro-inflammatory IFN-γ+ and IL17+ CD4 T cells in the spleen of NOD mice, while concurrently increasing the presence of regulatory IL-10+ and Foxp3+ CD4-T cells. This effect resulted in the mitigation of autoimmune insulitis (Duan et al., 2019). The imbalance in Teff/Treg ratios observed in collagen-induced arthritis (CIA) and Type 1 diabetes (T1D) is also evident in various other diseases, including T2D, obesity, aging, and rheumatoid arthritis.

## Relationship between the gut microbiota and metformin

**FIGURE 3:**
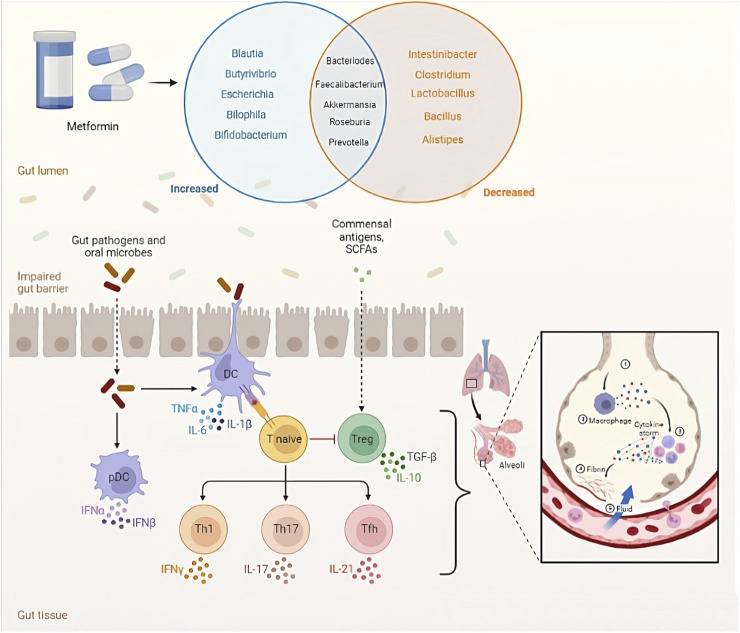
Microbiota-mediated effects of metformin.

Metformin is a widely used medication for the treatment of T2D that has been shown to improve insulin sensitivity, reduce blood glucose levels, and decrease the risk of cardiovascular disease (Kamyshnyi et al., 2021; Mohammed et al., 2021). Nevertheless, the precise mechanism of how metformin works remains incompletely understood. Lately, there has been increasing attention given to the connection between metformin and the gut microbiome. Several studies have indicated that metformin could potentially influence the gut microbiome, and these changes in the gut microbiome might be linked to metformin’s therapeutic effects (Table 2; Figure 3) (Forslund et al., 2015; Wu et al., 2017a; De La Cuesta-Zuluaga et al., 2017; Barengolts et al., 2018; Sun et al., 2018; Bryrup et al., 2019; Zhang et al., 2019; Chávez-Carbajal et al., 2020).

**TABLE 2:** Major findings from the research of patients with T2D with/without metformin treatment.

Sample size	Year of publication	Age	Techique	Associated microbiota changes	References
6 T2D with obesity	2020	47.0 ± 4.5 years	Whole-genome metagenomics shotgun	↓*Firmicutes, Oribacterium*, and *Paenibacillus*	Wang et al. (2020)
6 Controls					
183 T2D	2012	13– years	Metagenomic sequencing	↑*Akkermansia muciniphila, Bacteroides caccae, Clostridium hathewayi, Clostridium ramosum, Clostridium symbiosum, Desulfovibrio* spp*., Eggerthella lenta,* and *Escherichia coli*	Qin et al. (2012)
185 Controls				↓*Eubacterium rectale*, *Faecalibacterium prausnitzii*, *Roseburia intestinalis*, and *Roseburia inulinivorans*	
				Healthy controls had a ↑ abundance of butyrate-producing bacteria	
53 TD	2013	69–72 years	Metagenomic sequencing	↑ *Clostridium clostridioforme, Lactobacillus* spp*.,* and *Streptococcus mutans*	Karlsson et al. (2013)
49 Impaired glucose tolerance				↓ *Roseburia* and *Clostridium* spp., *Eubacterium eligens*, *Bacteroides intestinalis*	
43 Controls					
T2D with good glycemic control (52 patients)	2020	T2D with good glycemic control (66.38 ± 1.314 years)	16S rRNA sequencing	RT2D ↑ *Bacteroides vulgatus* and *Veillonella denticariosi*	Shih et al. (2020)
Refractory T2D (RT2D) (27 patients)		RT2D (64.37 ± 2.194 years)		RT2D ↓ *Akkermansia muciniphila* and *Fusobacterium* spp.	
Newly diagnosed T2D (50 patients) 50 Controls	2018	51 ± 12 years	16S rRNA sequencing	↑ *Lactobacillus* spp.	Chen et al. (2019)
				↓ *Clostridium leptum* and *Clostridium coccoides*	
134 Prediabetic	2018	57–68 years	16S rRNA sequencing	↓ *Akkermansia muciniphila* and *Clostridium* spp.	Allin et al. (2018)
134 Controls					
Treatment-naïve T2D (77 patients)	2019	61.75 ± 9.09 years	Whole-genome metagenomics shotgun	↑ *Escherichia coli, Eggerthella* spp*., Streptococcus salivarius*	Zhong et al. (2019)
80 Prediabetic				↓ *Clostridium* spp*., Faecalibacterium prausnitzii*	
97 Controls					
18 T2D	2010	31–73	16S rRNA sequencing	↑ *Bacteroidetes, Clostridium coccoides, Firmicutes*	Larsen et al. (2010)
18 Controls				↓ Proportions of phylum *Firmicutes* and class *Clostridia*	
40 T2D	2016	40–77	16S rRNA sequencing	↑ *Collinsella* spp*., Streptococcus* spp*., Lactobacillus* spp.	Candela et al. (2016)
13 Controls				↓ *Bacteroides* spp.*, Prevotella* spp., *Roseburia* spp., *Faecalibacterium* spp.	
25 T2D	2021	T2D (62.52 ± 7.58)	16S rRNA sequencing	↑ *Bifidobacterium* spp. and *Lactobacillus* spp.	Huang et al. (2021)
25 TD2 with retinopathy		Controls (57.8 ± 10.06)		↓*Escherichia-Shigella*, *Faecalibacterium*, *Eubacterium_hallii*_group and *Clostridium* genera	
25 Controls		TD2 with retinopathy (60.28 ± 10.5)			
Metformin-treated (MT) 93	2015	35–75 years	Metagenomic	↑*Escherichia* spp., ↓*Intestinibacter* spp.	Forslund et al. (2015)
Metformin-untreated (MUT) 106					
MT 14	2017	18–62 years	16S rRNA sequencing	↑ *Prevotella* spp., *Megasphaera* spp., *Butyrivibrio* spp	De La Cuesta-Zuluaga et al. (2017)
MUT 14				*Akkermansia muciniphila*	
				↓ *Oscillospira* spp., Barnesiellaceae	
27 healthy young men without T2D	2019	18–35 years	16S rRNA sequencing	↑ *Escherichia/Shigella* spp., *Bilophila wadsworthia*	Bryrup et al. (2019)
				↓*Intestinibacter* spp., *Clostridium* spp.	
Treatment-naïve T2D (22 patients)	2017	NA	Metagenomic	↑*Pectobacterium* spp., *Pantoea* spp., *Serratia* spp., *Dickeya* spp., *Helicobacter* spp., *Cronobacter* spp., *Erwinia* spp., *Yersinia* spp., *Enterobacter* spp., *Citrobacter* spp., *Escherichia* spp., *Bacillus* spp	Wu et al. (2017a)
Treatment-naïve T2D with metformin treatment 4 months (22 patients)				↓ *Dethiosulfovibrio* spp., *Deferribacter* spp., *Subdoligranulum* spp., *Intestinibacter* spp.	
Treatment-naïve T2D (22 patients)	2018	NA	Metagenomic	↓*Bacteroides fragilis*	Sun et al. (2018)
Treatment-naïve T2D with metformin treatment 3 days (22 patients)					
MT 21	2018	35–70	16S rRNA sequencing	↑*Bifidobacterium* spp., *Catenibacterium* spp., *Parabacteroides* spp.	Barengolts et al. (2018)
MUT 11					
T2D patients before MT 26	2019	NA	16S rRNA sequencing	↑ *Spirochaete* spp., *Turicibacter* spp., and *Fusobacterium* spp.	Zhang et al. (2019)
T2D patients after 3 months of MT 51					
Treatment-naïve T2D (14 patients)	2020	48.1 ± 4.7	16S rRNA sequencing	↑*Pelomonas* spp.	Chávez-Carbajal et al. (2020)
Treatment-naïve T2D with metformin treatment 3 days (14 patients)					

Research has revealed that the use of metformin is linked to alterations in the relative abundance and diversity of specific gut bacterial taxa (Petakh et al., 2023e). For instance, certain studies have found that metformin use is associated with an increased relative abundance of the genus *Lactobacillus* and a decreased relative abundance of the genus *Bacteroides* (Lee et al., 2021). Additionally, other studies have indicated that metformin use is linked to an increased relative abundance of the genus *Akkermansia*, a genus known for its potential to enhance insulin sensitivity (Ke et al., 2021).

The exact mechanisms by which metformin modulates the gut microbiome and how changes in the gut microbiome contribute to metformin’s therapeutic effects are not fully understood. Several potential mechanisms have been proposed, including the reduction of pro-inflammatory cytokines and the increase of short-chain fatty acids (SCFAs) (den Besten et al., 2013). In particular, SCFAs can bind to G-protein-coupled receptors (GPCRs) such as free fatty acid receptor 3 (FFAR3) and free fatty acid receptor 2 (FFAR2), which are expressed on enteroendocrine L cells, causing the release of glucagon-like peptide-1 (GLP-1) and Peptide YY. These hormones regulate glucose metabolism and insulin secretion (Holz et al., 1993). In rodent studies, an increase in acetate production caused by changes in gut microbiota activates the parasympathetic nervous system, leading to an increase in insulin secretion in response to glucose and increased secretion of ghrelin. This creates a positive feedback loop, causing excessive eating (hyperphagia), increased fat storage, and ultimately obesity (Perry et al., 2016). Long-term delivery of propionate to the colon has been found to significantly reduce weight gain and accumulation of fat in the abdomen, and also prevents a decline in insulin sensitivity in overweight adults (Chambers et al., 2015).

Human studies have demonstrated the beneficial effects of short-chain fatty acids (SCFAs) on glucose homeostasis and insulin sensitivity. For example, one study investigated the impact of delivering propionate to the colon in overweight and obese individuals using inulin-propionate ester (IPE). The supplementation improved insulin sensitivity compared to a low-fermentable fiber control (cellulose) and led to increased secretion of GLP-1 and Peptide YY. Additionally, changes in gut bacterial composition and markers of systemic inflammation were observed, highlighting the significant physiological impact of raising colonic propionate delivery in humans (Ch et al., 2019).

Another pilot-and-feasibility trial utilized high-amylose maize-resistant starch modified with acetate and butyrate (HAMSAB), resulting in increased SCFA concentrations in stools and plasma, along with alterations in gut microbiota composition and function. Subjects with the highest SCFA concentrations exhibited better glycemic control, although glucose control and insulin requirements remained unchanged (Bell et al., 2022). These findings suggest that targeting the gut microbiota and its metabolites, particularly SCFAs, may hold therapeutic potential for treating metabolic disorders like obesity and diabetes.

Metformin has been extensively researched in both animal and human subjects, and the findings suggest that it can influence the secretion of gut hormones and increase glucose uptake and utilization in the human intestine (Pénicaud et al., 1989; Bailey et al., 1992; Wilcock and Bailey, 1994; Ma et al., 2004; Duca et al., 2015; Koffert et al., 2017). However, the precise mechanism by which metformin enhances gut glucose uptake and utilization remains unclear.

Some studies have reported that metformin reduces the activity of sodium-glucose transporter 1 (SGLT1) while increasing the recruitment of glucose transporter 2 (GLUT2) to the apical membrane of rat jejunum (Sakar et al., 2010). Meanwhile, other research indicates that metformin increases SGLT1 gene expression in the duodenum and jejunum, but has no significant effect on GLUT2 gene expression in the intestine (Lenzen et al., 1996). Tongzhi Wu et al. also investigated that metformin inhibits small intestinal glucose absorption, which may contribute to augmented GLP-1 secretion (Wu et al., 2017b).

One of the mechanisms that triggers GLP-1 release is glucose absorption in the small intestine (Kuhre et al., 2015). Glucose-induced GLP-1 release is triggered by various underlying mechanisms, but it seems that SGLT1 plays a dominant role (Par et al., 2012). SGLT1 mediates the uptake of glucose with Na^+^, which depolarizes the membrane and stimulates Ca^2+^ entry. This, in turn, leads to the secretion of GLP-1 (Gorboulev et al., 2011; Kuhre et al., 2015). SGLT1’s dominant role in glucose-stimulated GLP-1 secretion is further supported by the fact that SGLT1^−/−^ mice not only show impaired glucose absorption but also impaired GLP-1 release (Gorboulev et al., 2012).

In studies using germ-free mice as a “microbial knockout” model, the transplantation of gut microbiota from healthy mice led to modifications in genes related to glucose metabolism in the gut (El Aidy et al., 2013). Prebiotics and probiotics have also been found to influence the gut microbiome, affecting GLP-1 secretion (Ejtahed et al., 2012; Balakumar et al., 2018). Bauer et al. demonstrated that metformin can alter the gut microbiome in the upper small intestine, resulting in an increase in SGLT-1 expression. In rodents fed a high-fat diet, SGLT-1 expression was reduced but could be restored with metformin administration (Bauer et al., 2018).

Research has shown that the presence of *Lactobacillus* significantly increases after metformin treatment, suggesting a possible link between *Lactobacillus* and the modification of SGLT-1 following metformin administration. This increase in SGLT-1 mediated metabolites produced by *Lactobacillus* has been found to enhance glucose uptake in Caco-2 cells, supporting the idea that *Lactobacillus* may be involved in the regulation of glucose metabolism influenced by metformin (Rooj et al., 2010). Thus, these findings indicate that *Lactobacillus* might play a role in regulating glucose metabolism and may be associated with improvements in glucose levels in both rodents and humans taking probiotic supplements containing *Lactobacillus* (Yadav et al., 2007). However, the specific mechanism by which metformin alters the amount of *Lactobacillus* in the gut remains unknown.

In conclusion, the relationship between the gut microbiome and metformin is an emerging field of research. While some studies have shown that metformin use is associated with changes in the gut microbiome, the exact mechanisms by which metformin modulates the gut microbiome and how changes in the gut microbiome affect metformin’s therapeutic effects are not yet fully understood and require further investigation.

## Interplay between metformin, bile acids, gut microbiome, GLP-1, and glycemic control in diabetes

Bile acids, traditionally known for their role in fat digestion and absorption, have been found to act as signaling molecules, influencing blood glucose regulation. When administered to different parts of the gastrointestinal tract, bile acids increase plasma GLP-1 concentrations and attenuate the blood glucose response to small intestinal glucose infusion in both healthy individuals and those with T2D (Wu et al., 2013a; Wu et al., 2013b; Brønden et al., 2017). The glucose-lowering effect of bile acids is believed to be mediated by the GLP-1 receptor, as evidenced by the inhibition of this effect in T2D patients treated with a GLP-1 receptor antagonist (Sansome et al., 2020).

Roux-en-Y gastric bypass surgery, a type of bariatric surgery, enhances GLP-1 secretion and metabolic improvements by diverting bile from the duodenum to the distal small intestine (Larraufie et al., 2019; Madsen et al., 2019). Researchers are exploring bile acid-based therapies that could mimic the effects of bariatric surgery by delivering bile acids to the distal gut, which holds promise for managing T2D (81).

Bile acids can interact with different receptors, including Takeda G-protein-coupled receptor 5 (TGR5) and farnesoid X receptor (FXR), both expressed in L-cells (Makishima et al., 1999; Ding et al., 2015). TGR5 activation by bile acids increases GLP-1 secretion, while FXR activation has a more variable effect (Thomas et al., 2009; Li et al., 2013; Trabelsi et al., 2015; Kuhre et al., 2018). Additionally, bile acids can activate bitter taste receptors throughout the gastrointestinal tract, leading to GLP-1 secretion and weight loss in rodents (Dotson et al., 2008).

Bile acid sequestrants have been investigated as a therapy for T2D (Hansen et al., 2017). Although they moderately reduce blood glucose, they also decrease GLP-1 secretion when combined with exogenous or endogenous bile (Adrian et al., 2012; Hansen et al., 2016; Brønden et al., 2018). The exact role of this reduction in GLP-1 in the benefits of bile acid sequestrants remains unknown, necessitating further research on their chronic administration.

Metformin inhibits bile acid resorption, resulting in increased fecal bile salt excretion (Scarpello et al., 1998). This mechanism may explain the gastrointestinal adverse effects, such as diarrhea, associated with metformin (Watson et al., 2019). Moreover, by reducing proximal bile acid reabsorption, metformin increases the exposure of the distal gut to bile acids, potentially enhancing bile acid-induced GLP-1 secretion and glucose-lowering effects (Sansome et al., 2020).

The gut microbiota, a group of bacteria in the gastrointestinal tract, can be disrupted, leading to dysbiosis and contributing to various diseases like obesity, T2D, and allergies (Gomaa, 2020). Both metformin administration and T2D have been associated with changes in gut microbial composition (Qin et al., 2012; Forslund et al., 2015). Clinical trials have shown that metformin administration can lead to changes in several bacterial strains in healthy individuals and those with T2D (Wu et al., 2017a). These changes may improve glucose tolerance and insulin sensitivity. Notably, metformin use is linked to the reduction of *Bacteroides fragilis* and alterations in bile acid composition, which can enhance GLP-1 secretion and inhibit FXR activity (Sun et al., 2018). Although the specific bacterial strains affected may vary between studies, it is evident that metformin independently influences the gut microbiota regardless of T2D presence (Sansome et al., 2020).

## The role of gut microbiota in SARS-CoV-2 infection

SARS-CoV-2, the causative agent of COVID-19, has been shown to infect the gastrointestinal tract, and alterations in gut microbiota composition have been linked to an increased susceptibility to viral infections (Li et al., 2022; Xiang and Liu, 2022). Studies have reported a decrease in beneficial gut bacteria, such as *Faecalibacterium prausnitzii* and *Bifidobacterium*, and an increase in pathogenic bacteria, such as *Enterococcus faecalis* and *Streptococcus*, in COVID-19 patients (Yeoh et al., 2021; Ancona et al., 2023; Petakh et al., 2023a). These changes in gut microbiota composition were associated with increased levels of inflammatory markers, such as IL-6 and CRP (Petakh et al., 2023a; Gradisteanu et al., 2023). Moreover, COVID-19 patients with gastrointestinal symptoms had a higher abundance of opportunistic pathogens, suggesting that gut microbiota dysbiosis may contribute to the severity of gastrointestinal symptoms in COVID-19 patients (Wang et al., 2022).

The gut microbiota can influence the host’s immune response to viral infections by regulating the production of antiviral cytokines and modulating the activity of immune cells. For example, gut bacteria can produce SCFAs, which have been shown to enhance the antiviral immune response by increasing the production of IFN-γ and natural killer (NK) cells (Carreca et al., 2022; Govers et al., 2022). Moreover, the gut microbiota can influence the development and function of Tregs, which play a crucial role in maintaining immune homeostasis and preventing excessive inflammation (Smigiel et al., 2014).

Recent research indicates that the gut microbiota may play a role in influencing the immunogenicity of COVID-19 vaccines (Ng et al., 2023). In one study, the use of antibiotics before vaccination was associated with lower seroconversion rates and median antibody levels after receiving one dose of the BNT162b2 vaccine, although this effect was not observed after receiving two doses (Che et al., 2022). Although the study did not directly analyze fecal microbiota, it suggests that dysbiosis in the gut microbiota might have influenced the immune response to the COVID-19 vaccine.

A study involving patients with inflammatory bowel disease found that those with below-average concentrations of SARS-CoV-2-specific antibodies had lower gut microbiota beta diversity and exhibited different bacterial abundances compared to those with above-average antibody concentrations (Alexander et al., 2023). Additionally, differential abundance of fecal metabolites was observed in above- and below-average responders. Specific gut microbial species, such as Bilophila, were associated with above-average response, while others like *Streptococcus* were associated with below-average response. These findings imply that certain gut microbial species and metabolites, including trimethylamine, short-chain fatty acids (SCFAs), and bile acids, could be linked to COVID-19 vaccine immunogenicity. However, it is essential to consider that these studies have limitations, such as small sample sizes and the lack of analysis of other factors that might influence the gut microbiota and metabolome.

In addition to the gut microbiota’s role in modulating the immune response to viral infections and influencing COVID-19 vaccine immunogenicity, emerging evidence suggests the existence of a gut-lung axis that may contribute to the pathogenesis of COVID-19. The gut-lung axis represents a bidirectional communication pathway between the gut and the lungs, where alterations in the gut microbiota can affect lung health and *vice versa* (Synodinou et al., 2022).

Studies have demonstrated that gut dysbiosis, characterized by an imbalance in the gut microbial composition, can lead to systemic inflammation and immune dysregulation, which may have implications for lung diseases. In the context of COVID-19, it has been proposed that the gut-lung axis could influence the severity of respiratory symptoms and the risk of developing complications (Petakh et al., 2023a; Petakh et al., 2023c; Petakh et al., 2023d).

Metabolites produced by the gut microbiota, such as SCFAs and bile acids, have the ability to enter the bloodstream and exert effects on distant organs, including the lungs. SCFAs, generated by certain gut bacteria through the fermentation of dietary fiber, possess immunomodulatory properties and can influence immune responses in the lungs (Dang et al., 2019). They are known to regulate the production of inflammatory cytokines and promote the generation of regulatory T cells, which help maintain immune balance and reduce excessive inflammation in the lungs (de Oliveira et al., 2021).

Moreover, alterations in the composition of the gut microbiota can lead to increased gut permeability, enabling the translocation of bacterial components, such as lipopolysaccharides (LPS), into the bloodstream. This process, referred to as bacterial translocation, can trigger systemic inflammation and contribute to the development of lung injury (Sun Z. et al., 2022). In COVID-19 patients, the presence of circulating LPS has been associated with disease severity and poorer clinical outcomes (Giron et al., 2021).

## Conclusion

In conclusion, metformin has shown potential as an antiviral agent against SARS-CoV-2, as well as other RNA viruses. It may inhibit viral entry into cells and suppress viral growth in cell culture models. Clinical trials have demonstrated promising results, with metformin leading to a decrease in viral load and a higher rate of undetectable viral load in COVID-19 patients. Furthermore, metformin’s anti-inflammatory effects may help prevent cytokine storms by reducing the production of pro-inflammatory cytokines and modulating immune cell function. The drug’s ability to regulate Th17/Treg balance and influence immunometabolism suggests its potential in mitigating inflammation and restoring T cell functionality in COVID-19. Additionally, metformin’s modulation of the gut microbiota, particularly changes in bacterial taxa and the production of short-chain fatty acids, may contribute to its therapeutic effects. Bile acids, gut microbiota, and their interplay with metformin and GLP-1 have implications for glycemic control and the management of diabetes. Understanding the relationship between metformin, the gut microbiome, and SARS-CoV-2 infection opens new avenues for research and potential therapeutic interventions in COVID-19.
